# Thoracodorsal Artery Perforator Flap for Chronic Radiation-Induced Ulcer of the Axilla in Vietnam

**DOI:** 10.1155/2021/8478006

**Published:** 2021-10-06

**Authors:** Hong Quang Le, Anh Dung Hoang

**Affiliations:** Department of Breast Surgery, Vietnam National Cancer Hospital, Hanoi, Vietnam

## Abstract

Breast cancer is the leading cause of death in females worldwide. Radiotherapy plays an important role for locoregional control in the comprehensive management of breast cancer. Chronic radiation-induced ulcer of the axilla can occur, and it is complicated to treat for these lesions. The application of a thoracodorsal artery perforator flap offers many advantages to be one of the most efficient treatments for radiation-induced ulcers of the axillary region. We introduce a series of 5 patients with radiation-induced ulcers of the axilla treated by using a thoracodorsal artery perforator flap. The mean operative time was 190 minutes. During at least a two-year follow-up, no complication has been found, and the patient has achieved good cosmetic result without movement limitation of the upper limb.

## 1. Introduction

Breast cancer is a common cause of death in females worldwide. According to GLOBOCAN 2018, there were more than 2 million new cases and above 600,000 deaths due to breast cancer, in which Vietnam recorded 15,000 new cases and 6,000 deaths [1].

The treatment of breast cancer is multidisciplinary, and radiotherapy plays an important role for locoregional control. Radiotherapy helps to make surgery less extensive while maintaining relapse-free and overall survival rates [2]. However, radiation therapy is associated with long-term adverse events that compromise patients' quality of life. Radiation-induced ulcer is caused by the acute or chronic effects of ionizing radiation. When the lesion becomes chronic, it is difficult to heal due to lack of blood supply, fibrosis, and impaired cellular repair capacity. The injury may involve the skin, underlying soft tissue, and even deep structures such as bone, which can appear many years after radiation treatment. In developing countries like Vietnam, modern radiation techniques such as IMRT and VMAT are not widely used and the burden of radiation-induced adverse effects is high.

The treatment of chronic radiation-induced ulcers in the chest wall (CRUCW) is complicated since it is often associated with radiation osteomyelitis, radiation pneumonitis, brachial plexus injury, and other comorbidities. Besides, these ulcers might cause fibrosis of the axilla and movement limitation that have worse impact on patients' daily activities. Conventional surgical treatment methods such as thin skin flap, split-thickness skin graft, total skin flap, Z-plasty, or rotation flap have low efficacy. The discovery of perforator flaps is an advance of reconstructive surgery. A thoracodorsal artery perforator flap (TDAP) was used by Angrigiana et al. since 1995 that was called “latissimus dorsi musculocutaneous flap without muscle” [3]. Since 2005, other authors including Mun et al. [4] and Schaverien et al. [5] have conducted more thorough studies about anatomy and clinical application of the flap in the treatment of axillary ulcer and in reconstructive surgeries afterwards. A TDAP flap has many advantages such as not causing damage to the latissimus dorsi muscle, relatively constant blood vessels, longer blood supplying vessels with the use of a perforator branch, shorter time of recovery, and a relatively thin skin flap which is suitable for the axillary region. This work has been reported in line with SCARE criteria [6].

## 2. Presentation of Cases

We hereby report 5 cases of breast cancer patients who had surgical treatment of radiation-induced axillary ulcer by the TDAP flap from 01/2016 to 01/2019 at Vietnam National Cancer Hospital. These patients had Cobalt-60 external beam radiotherapy with the dose of 50 Gy. Details of their clinical features are summarized in Table 1. Before surgery, the patients' comorbidities were stabilized, culture of ulcer discharge was performed, and antibiotics were given accordingly until the ulcer became clinically clean. The biopsy of the lesion was taken for all patients to rule out local recurrence or secondary sarcoma due to radiation. They also had chest and abdominopelvic CT scan and bone scan to confirm that there were no metastases and radiation-induced ribs and clavicle lesions.

The patient was put under general anesthesia with endotracheal intubation and placed in a lateral position with the ulcer side facing upward. The ipsilateral arm was placed at a 90-degree angle to the axis of the body. Radical excision of the ulcerated lesion was performed until healthy tissue was present at the margin. The axillary fibrosis was dissected, and the operative field was sterilized with 10% iodine. The perforator branch was evaluated and marked with Doppler ultrasound before surgery. We then planned a flap reconstruction based on the location of the perforator branch and size of the ulcer. During the surgery, we harvested the flap and created a tunnel that the flap could be pulled through to the axilla followed by careful coagulation. The pedicle was checked to make sure that there was no tension or twist. We used two drain tubes, one in the flap harvested site and one in the axilla. Operative duration, number of perforator branches, length of stay, and postoperative complications including bleeding, infection, seroma, and flap necrosis were recorded. The patients were followed up at 3 months, 6 months, 1 year, and 2 years after surgery.

**Figure 1:**
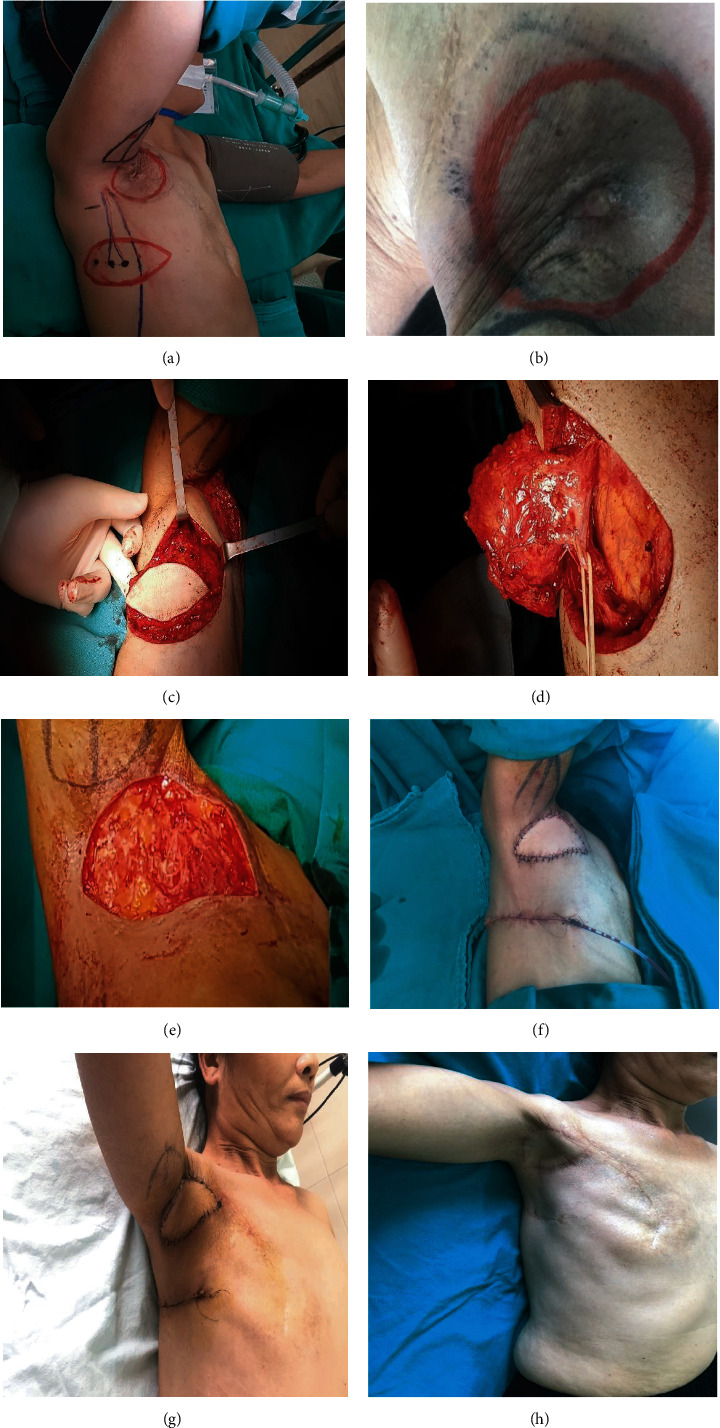
Patient no. 1: (a) surgical position; (b) preoperative lesion; (c, d) flap dissection; (e) excision of the lesion; (f) TDAP flap; (g) 3 weeks after surgery; (h) 2 years after surgery.

**Figure 2:**
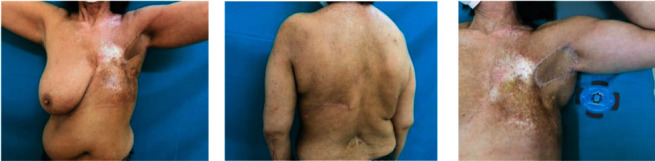
Patient no. 2: two-year postoperative result.

**Figure 3:**
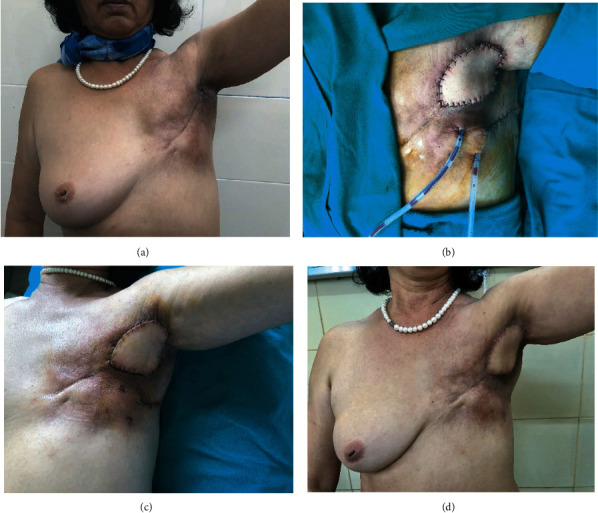
Patient no. 3: (a) preoperative lesion; (b) TDAP flap; (c) 3 weeks after surgery; (d) 2 years after surgery.

The mean operative time was 190 minutes, and the largest size of the flap was 14 × 8 cm (see details in Table 2). There were 3 cases with 2 perforator branches and 2 cases with 1 perforator branch. The duration of postoperative care was from 7 to 17 days. Most of the flaps were well vascularized, and there was only one case with marginal flap ischemia (patient no. 2 with diabetes). During follow-ups, the patients had good cosmetic results and no movement limitation was recorded. The operative procedure and cosmetic results are illustrated in Figures 1–3.

## 3. Discussions

Although radiotherapy plays an important role in the multidisciplinary treatment strategy of breast cancer, several adverse effects especially on skin have been reported. The prevalence of moderate to severe effects may come up to 85% [7], and radiation-induced ulcer is one of the most serious complications. In Vietnam, there is still a lack of reliable research on the prevalence of radiation-related ulcer. However, several typical cases have been recorded in clinical practice.

Unlike other skin ulcers which can recover with medical treatment, radiation-induced ulcer (RIU) is difficult to treat and significantly affect patients' quality of life. Ischemia, recurrent infection, and poor healing are the causes of unfavorable progression of the ulcer [8]. Radiation-induced ulcer could appear after 3 months to 25 years and is usually gradually progressive. In our 5 cases, the longest time from radiation treatment to ulcer presence was 20 years. The principle of RIU treatment is to completely remove all the irreversibly damaged tissue including skin, fat, muscle, and even bones [9]. To cover the wound, skin grafting has a low successful rate because of radiation-related ischemia and lack of oxygenation [10]. The use of local flaps is not encouraged since the surrounding tissue is also affected by radiotherapy [11]. Pedicle flaps from nearby sites which are out of the radiation field might be promising. With the development of microsurgery techniques, surgeons can choose a suitable flap which fits the size and shape of the lesion. However, finding a good vascular pedicle for a free flap is not easy since radiation-induced intravascular inflammation may cause thrombosis and increase the complication rate [12]. Moreover, microsurgery requires complicated facilities and techniques and has long operation time.

RIU in the axilla is often associated with fibrosis and movement limitation of the shoulder. Previous reports on RIU after breast radiotherapy mainly focused on chest wall ulcers, whereas there were limited data on axilla ulcers. The aim of the treatment of axilla RIU also includes recovering the movement range of the shoulder. Many nearby flaps such as scapular flap and parascapular flap, anterior arm flap, posterior arm flap, pectoralis major myocutaneous flap, intercostal flap, or latissimus dorsi muscle flap could be used to cover the lesion, but they have some limitations. For example, a scapular flap and parascapular flap have short pedicle and thick subcutaneous tissue, and the skin has different color compared to axilla skin [13]. An anterior arm flap, posterior arm flap, and intercostal flap have small size, and the color is also not suitable. A latissimus dorsi muscle flap is not thin enough to facilitate movement of the shoulder, and the function of the latissimus dorsi muscle is also affected.

In 2013, Yang et al. reported a satisfying outcome of 59 patients with chest wall and axilla ulcers who were treated with a TDAP flap from 04/2007 to 08/2011, in which 1-2 perforator branches were identified and the largest flap size was 11 × 15 cm and there was no blood vessel constriction or obstruction [14]. They concluded that the TDAP flap provided a reliable thin and large flap to cover the axilla, which was also muscle-sparing and did not significantly affect the muscle function. In our 5 patients, 4 patients had a well-vascularized flap. Patient no. 2 had marginal ischemia and required longer hospital stay (17 days) because there was only one perforator branch identified and she had severe diabetes. The wound where the flap was harvested was closed without skin grafting in all 5 patients, and there were no complications such as infections, necrosis, and especially seroma. The function of latissimus dorsi muscle was well preserved. A report of Hamdi et al. showed no differences of strength and thickness of the muscle and movement range of the shoulder compared to the contralateral side [15, 16].

Preoperative identification of the perforator branch is essential for the surgical plan to design a suitable flap. There are various methods such as Doppler ultrasound, CT scan [17], DSA, or MRI. However, most authors used Doppler ultrasound because of its convenience and high efficacy [18, 19]. Thanks to Doppler ultrasound, the mean operation time of our 5 patients was 190 minutes with 1-2 perforator branches dissected.

In reports on the utility of the TDAP flap, in most of cases, they are applied for covering the skin defect in the chest or axilla region of extremity after crush injury and burn contracture of some diseases such as hidradenitis suppurativa. In comparison with these cases, there are many complicated problems when using the TDAP flap for treating radiation-induced ulcer of the axilla: low potential of recovery in affected tissue, deep ulcer, low blood supplement, and irreversible process of radiation-induced fibrosis. In addition, the ulcer is at high risk of infection due to poor hygiene of the axilla region and lack of regular healthcare, especially in environmental conditions with hot and humid tropical climate like Vietnam, which is suitable for the survival, development, and spread of bacteria. Therefore, the ulcer needs to be carefully treated with medicine such as antibiotic and anti-inflammatory drugs before operation. Two cases of our patients were discovered to be infected with *P. aeruginosa*, and they were treated with antibiotic for three weeks to prepare for operation. It is essential to take a careful preparation, and identification of bacteria from the infected ulcer is very essential for a favorable postoperative outcome.

Similar to other surgical cancer centers in Vietnam, our hospital considers the latissimus dorsi flap a good choice in breast reconstruction and skin coverage for radiation-induced ulcer or burn contracture. However, this flap seems to be unsuitable for axilla ulcer since this bulky flap may reduce the range of shoulder joint movement significantly and thus may affect the quality of life consequently. In contrast, by periodic examination of five patients, we recorded that the depth and skin color of TDAP flaps were highly applicable to the axilla region. Moreover, all of these patients have achieved a good range of upper extremity movement. The results from five patients have contributed considerable clinical data and offer a favorable solution for treating radiation-induced ulcer in chest or axilla regions.

## 4. Conclusions

TDAP is a safe and suitable flap for the treatment of axilla RIU. The function of the latissimus dorsi muscle was well preserved. Our results warrant for further study with a larger sample size and longer follow-up.

## Figures and Tables

**Table 1:** Clinical characteristics and culture results of the patients.

No.	Age	Stage	Comorbidities	Time of radiotherapy (years ago)	Preoperative medical treatment duration	Discharge culture
1	66	T2N1M0	None	15	1 week	Negative
2	55	T3N1M0	Diabetes	3	3 weeks	*P. aeruginosa*
3	60	T3N2M0	None	20	1 week	Negative
4	50	T4bN2M0	Diabetes	1	3 weeks	*P. aeruginosa*
5	70	T2N2M0	None	5	2 weeks	Negative

**Table 2:** Surgical results of the patients.

No.	Operative time (minutes)	Postoperative stay (days)	No. of perforator branches	Flap size (cm)	Complications	Duration of follow-up (years)
1	200	7	2	14 × 8	No	3
2	190	17	1	12 × 6	Marginal ischemia	2
3	160	8	2	13 × 5	No	2
4	205	10	2	10 × 5	No	2
5	185	7	1	12 × 6	No	4

## Data Availability

The data of the current study is available from the corresponding author on reasonable request.
